# Kansei Engineering in the Evolving Service Sector: A Decade of Insights

**DOI:** 10.12688/f1000research.174681.2

**Published:** 2026-06-03

**Authors:** Markus Hartono, Christabel Parung

**Affiliations:** 1 Industrial Engineering, University of Surabaya, Surabaya, Indonesia; 2 Department of Product Design, University of Surabaya, Surabaya, 60293, Indonesia

**Keywords:** Customer satisfaction, emotional needs, Kansei Engineering, service design

## Abstract

**Background:**

Kansei Engineering (KE) has increasingly been applied beyond product design into service contexts, responding to the growing importance of emotional satisfaction, experiential quality, and human-centered service design. Despite its expanding use, a comprehensive understanding of how KE has evolved methodologically, theoretically, and contextually within service research remains limited. This study aims to critically review KE applications in services over the last decade to identify key trends, contributions, and research gaps.

**Methods:**

A semi-systematic literature review was conducted using a two-phase Define–Refine protocol. A structured search was performed using the Scopus database covering publications from 2010 to 2023. The review followed PRISMA-guided screening and refinement procedures, resulting in the selection of 28 peer-reviewed journal articles. The selected studies were analysed through iterative thematic synthesis, methodological comparison, and cross-industry analysis.

**Results:**

The findings reveal four major thematic clusters of KE applications in services: (1) KE for service quality enhancement, (2) data-driven and analytics-based KE, (3) KE in digital and smart service systems, and (4) behavioral and psychophysiological KE. The review demonstrates a significant methodological shift from traditional attribute–response models toward more data-driven and computational approaches, including text mining, machine learning, sentiment analysis, and advanced statistical modelling. In addition, KE increasingly serves as an integrative framework, combining service quality models, decision-support systems, and intelligent service technologies. A cross-sector comparison further reveals varying levels of methodological maturity across logistics, hospitality, transportation, healthcare, and digital services.

**Conclusions:**

This study provides both theoretical and practical insights into the evolving role of KE in service research and development. By mapping the methodological evolution and thematic diversification of KE applications, the review highlights the growing importance of emotionally informed, data-driven, and human-centered approaches in contemporary service design. The findings also identify emerging opportunities for integrating KE with artificial intelligence, adaptive systems, and culturally sensitive service innovation. The study is limited by its reliance on a single database and the interpretive nature of the semi-systematic review approach.

## I. Introduction

Basically, in most countries, the service industry is an important contributor to their gross domestic product (GDP). A service industry is deemed to be a supporting activity that adds value to the existing products and processes. For instance, financial services can help banking customers manage their funds more efficiently and effectively. Over the past 20 years, the average contribution of services to GDP and value added has increased, leading to a stronger correlation between service expansion and overall economic growth. Value added from services made up 74% of GDP in high-income nations in 2015, up from 69% in 1997. ^1^ The United States' value-added contribution to GDP from services was higher than that of other high-income peer nations. From an average of 48% in 1997 to 57% in 2015, the GDP share of services in low- and middle-income nations increased even further. In 2021, the service sector accounted for about 78% of the US GDP. ^2^ Retail, banking, aviation, education, hospitality, healthcare, and entertainment are a few examples of the services that may fall under this category.

Other nations, such as those in Asia, are also witnessing an increase in the service sector's GDP contributions. In other words, it is stressed that this trend is not only limited to the United States. People typically expect more services and a higher quality of life as GDP and living standards rise. In Asia, the service sector provides both promising and challenging opportunities. Services are now a major driver of the region's output, growth, and employment. However, transitioning from old and low-value operations to modern and high-value activities is considered Asia's largest service industry problem. ^3^ Inherently, there are two major sectors that need to shift into the service economy in developed countries, which are agriculture and manufacturing. As economies mature, more people run businesses in the service sector. However, in emerging economies, the service sector tends to grow as income levels rise, and customer demands change. There might still be an important contribution from the manufacturing and agriculture sectors. Nevertheless, service research still plays a critical role in understanding and shaping the evolving landscape of how services are delivered and experienced. ^4^ Technological advances, especially in information technology, have transformed service interactions, leading to new ways for customers to engage with services before, during, and after purchase. The more satisfied customers, the better the quality of service is.

In capturing and modelling service quality, Parasuraman et al. ^5^ have developed the SERVQUAL model to assess service quality in various organizations over the last 30 years. Numerous authors have examined, critiqued, and modified the SERVQUAL questionnaire to meet the requirements and peculiarities of various service businesses. Still, it hasn't changed all that much as a standalone tool. Researchers have demonstrated the relevance and utilization of the SERVQUAL model for service quality improvement and innovation, considering relevant methods and service settings.

Apart from cognitive interaction, customers and service interactions should have an emotional bond. Most services are experiential and intangible. It is not possible to store or exchange services. Kansei Engineering (KE) has shown a profound understanding of the relationship between customer satisfaction and service delivery, particularly in terms of positive emotional responses and impressions. KE assists managers and service providers in providing experiences that spark favorable feelings and build emotional bonds with clients. We anticipate an increase in customer loyalty, positive word-of-mouth recommendations, and repeat business. Cognitive satisfaction with customers will increase. Affective satisfaction follows suit. Customers will therefore proportionately and simultaneously gain both cognitive and affective satisfaction. ^6^

Once their emotional needs are satisfied, customers are more likely to consider and perceive the service offers as advantageous and fulfilling. By providing distinctive and incredibly moving experiences that others might not be able to offer, KE sets itself apart from other comparable approaches in terms of creative approaches to service design and delivery. With KE, design is more focused on the affective needs of the user (also known as Kansei), making sure that products and services are made with the ideas and emotions of potential customers in mind. Since the 1970s, KE has been used and implemented in a variety of manufactured goods and other tangible goods. Since then, the use of KE has grown to encompass artificial intelligence (AI)-based services as well as service industries.

Since 2010, the use of KE in service sectors has been extensively documented in book chapters and international publications. Restaurants, banking, healthcare, logistics, and higher education were among their service sectors. to evaluate each conceptual and application framework's effectiveness, find gaps in the quality of various service settings, and offer conceptual and application frameworks. There has been extensive research on KE in services. ^6^ Although value creation is becoming more closely linked to customer experience and affective engagement, service industries still account for most of the global economic development. While SERVQUAL and other traditional service quality models have placed a strong emphasis on cognitive evaluation, modern service ecosystems demand a deeper comprehension of psychological, emotional, and sensory reactions. Although KE offers an organized method for simulating customer emotions, its use in service settings is still uneven, disjointed, and conceptually undeveloped. This review addresses three significant gaps in Knowledge Engagement (KE) literature within service contexts: (1) The lack of integrative theoretical synthesis connecting KE with related theories such as affective design and service-dominant logic; (2) Fragmented methodological development spanning traditional models to AI-driven analysis without critical comparison; and (3) Insufficient cross-sectoral insights contrasting KE performance in diverse fields like hospitality, logistics, healthcare, and e-commerce. The review aims to analyze KE applications from 2010 to 2023, offering both descriptive and interpretive insights grounded in theory and methodology.

However, there isn't a comprehensive study or paper that enumerates, organizes, and assesses all relevant work related to recommended KE frameworks, methodologies, and service implementation. To identify trends and develop themes in KE services, we conducted a mixed qualitative and quantitative literature evaluation. All relevant articles published this decade also cover the status of KE's services.

This paper points out the importance of research into KE in services as a specific field and provides the reader with a comprehensive context for understanding ongoing research. A body of KE knowledge in services will be reviewed for key findings and potential research gaps to help the researcher formulate potential research questions. Researchers and practitioners find this blended literature evaluation to be beneficial when trying to connect and evaluate many studies on various topics, either for interconnectivity or reinterpretation. ^7^ It is therefore a useful tool for producing theories and hypotheses. It will also add value by presenting a comprehensive and well-structured summary of the literature on KE in services and drawing perceptive conclusions.

## II. Brief of Kansei engineering

### A. Kansei and Kansei engineering

The literature review on KE in services aims to systematically gather, analyze, and critically interpret various studies of Kansei Engineering (KE) proposed and applied to service sectors. Nowadays, one of the most prominent economic sectors is that of services. This study uses evidence-based literature to mitigate potential biases and methodically integrates and organizes prior KE in-service research to provide clearer insights. Inherently, KE has various definitions. KE is an engineering approach to product and service design that considers the emotional needs of customers. It is a design methodology that attempts to understand, capture, and translate user feelings and emotions into the parameters of goods and services. The strategy is built based on the idea that people's emotional experiences have a significant impact on their long-term use of products and services. It is also deemed a multidisciplinary approach to product and service design and development that seeks to gather the emotional and psychological responses of users to product features, design, and aesthetics. ^8^ The method combines engineering, design, and psychology approaches to produce services and goods that are not only useful but also elicit desired feelings.

KE has been applied extensively in the fields of information technology, fashion, product design, and automotive design. For example, in the automotive design industry, KE has been applied to design cars that evoke emotions and impressions, like sportiness, tightness, or luxury. In the electronics and appliances, this method has been used to satisfy users’ functional needs while simultaneously indulging positive emotions like relaxation, excitement, or joy. ^9^ In general, KE usually comprises several steps, such as determining the target emotions (known as Kansei words), choosing the product domains that affect those emotions, building prototypes, and conducting user testing to confirm that the design is fit to the intended emotions.

Research has shown that the application of KE can result in enhanced customer satisfaction and product design. It may help create more appealing and enjoyable products and services. Further, this will lead to customer satisfaction and loyalty. In other words, this approach carefully considers how users will emotionally and psychologically respond to the appearance, features, and attributes of a product or service. ^10^ KE has evolved from its original focus on quantifying emotional impressions into a multifaceted design methodology that integrates various theoretical frameworks within service contexts. By aligning with affective design theory, the psychology of emotion, service-dominant logic, and the experience economy, KE enhances the understanding and design of emotional value in service experiences. This shift from a simple emotion-to-attribute mapping to a more complex emotion-to-experience-to-behavior pathway reflects the growing importance of experiential design and behavioral research in creating impactful customer journeys. Again, it not only fulfils functional requirements but also sparks positive emotions and feelings, which is beyond rational satisfaction.

### B. The application of Kansei engineering in services

Research and development on the use of KE in services has been ongoing recently. It has been intensively and extensively applied in both product and service sectors to boost customer satisfaction, experiences, and loyalty. Key uses of KE in services include the following: ^6, 8, 10^

• KE supports innovative service design. By applying this method, services that better address customers’ emotional and psychological needs can be proposed and implemented. The strategy is capturing and finalizing the emotions that customers connect with various attributes of a service, then utilizing that knowledge to create a service that more effectively satisfies their emotional needs.
• KE plays a crucial role in customer experience management. By adding sensory and emotional design elements to the service, KE can be used to enhance customer experiences. For instance, using music, ambient lighting, and other sensory cues can make a customer's experience more enjoyable and memorable.
• The focus of KE is on customer emotional satisfaction. Implementing the emotional and sensory components of services importantly demonstrates and quantifies customer emotional satisfaction.
• KE leads to customer loyalty. By addressing a more emotionally fulfilling customer interaction, KE can be used as a catalyst of customer loyalty. According to a previous prominent study (see ^11^), for instance, customers who received services created with significant Kansei were more likely to be loyalists.

Based on the backbone of research, KE may have an important impact on design and service delivery. This strategy can be very helpful to service designers and managers because it has been shown to improve customer satisfaction, experience, and loyalty. Further research is required to fully understand the potential of this method in the services sector and to identify best practices for its application. Numerous service sectors, such as hospitality, tourism, retail, and healthcare, have used KE. For instance, KE has been applied to the hospitality industry to better design restaurant and hotel rooms that cater to the emotional and sensory needs of patrons. KE has also been used in the retail industry to design store environments that give customers a more enjoyable and emotionally engaging shopping experience. One of KE’s main advantages for service providers is that it makes their offerings more distinctive and memorable for customers.

### C. Kansei and Servqual

To enhance service quality, as previously discussed, KE can also be used in the design and development of services. The method is used in service design to understand how clients react emotionally and psychologically to different elements of the service, including the setting, interactions with staff, and the overall service experience. In service design, KE targets not only satisfying functional needs but also simultaneously evoking favorable customer delights. Increased client happiness, loyalty, and general service quality are the target.

When utilizing KE for service design, there are usually more than one phase. These steps are necessary to make sure that the design makes people feel the way you want them to, as follows. Figuring out what feelings you want to elicit, choosing service qualities that will affect them, making prototypes, and testing with consumers. Research shows that integrating KE into service designs may make the service better, make customers happier, and make them more loyal. KE helps make services more fun and interesting to use, which makes customers happier and more loyal. KE does this by considering how customers feel and what they want while designing.

### D. Challenges and opportunities of Kansei engineering

The service business quickly adopts new technology to improve customer happiness. It might result in higher output and efficiency, as well as the development of new services. Everybody helps the economy grow. It impacts the Kansei, bringing up matters of loyalty and trust. The differences in culture are another factor. KE can assist services in adjusting to cultural quirks and guaranteeing that emotional bonds are both appropriate and productive in the target culture. Furthermore, maintaining faithfulness over an extended period is beneficial. KE cultivates strong emotional ties with its customers, which over time may result in continued engagement and loyalty. Figuring out how service design features connect to customers' feelings is a key issue for KE when providing services. It guides service firms in developing marketing strategies and inspiring innovative service design concepts. According to, ^12^ customers are continuously looking for new services after experiencing the ones they have now. Thus, the concept of fresh service design is never-ending. Designers must always understand the true feelings of their clients to build a new service that meets their expectations.

## III. Objective of the study

With a primary focus on Kansei (customer emotional needs), this study analyses KE research and its application in services over the past ten years and provides a framework for thinking about future research directions in service innovation and development.

This study critically evaluates the application of KE in service sectors. It aims to identify KE's theoretical and methodological trajectories, compare its applications across industries for effectiveness, develop a conceptual framework for KE-enabled service innovation, and propose future research pathways based on empirical and theoretical gaps.

## IV. Methodology

Some of the most important approaches based on KE and their main findings in the fields of service design and innovation, as well as services in general, are discussed in this paper. A semi-systematic literature review was adopted as the methodological approach in this study. This approach is particularly appropriate for research areas that are multidisciplinary, conceptually diverse, and characterized by heterogeneous methodologies. ^13^ KE research spans multiple domains, including engineering, design, psychology, and service science, resulting in fragmented and variably defined bodies of knowledge. According to Hanna Snyder, ^14^ semi-systematic reviews are suitable for synthesizing broad and evolving research areas, where the objective is not to answer narrowly defined research questions but to map key themes, theoretical developments, and research trajectories over time. In contrast to strictly systematic reviews, which rely on rigid inclusion criteria and predefined protocols, semi-systematic reviews allow a more flexible, interpretive approach that accommodates conceptual diversity and methodological variation.

In this study, the semi-systematic approach enables the integration of both qualitative and quantitative research on KE in service contexts, supporting a comprehensive understanding of its development over the past decade. The review was carried out in two stages, and it was presented as an efficient way to evaluate several research articles or publications.

**Figure 1.**
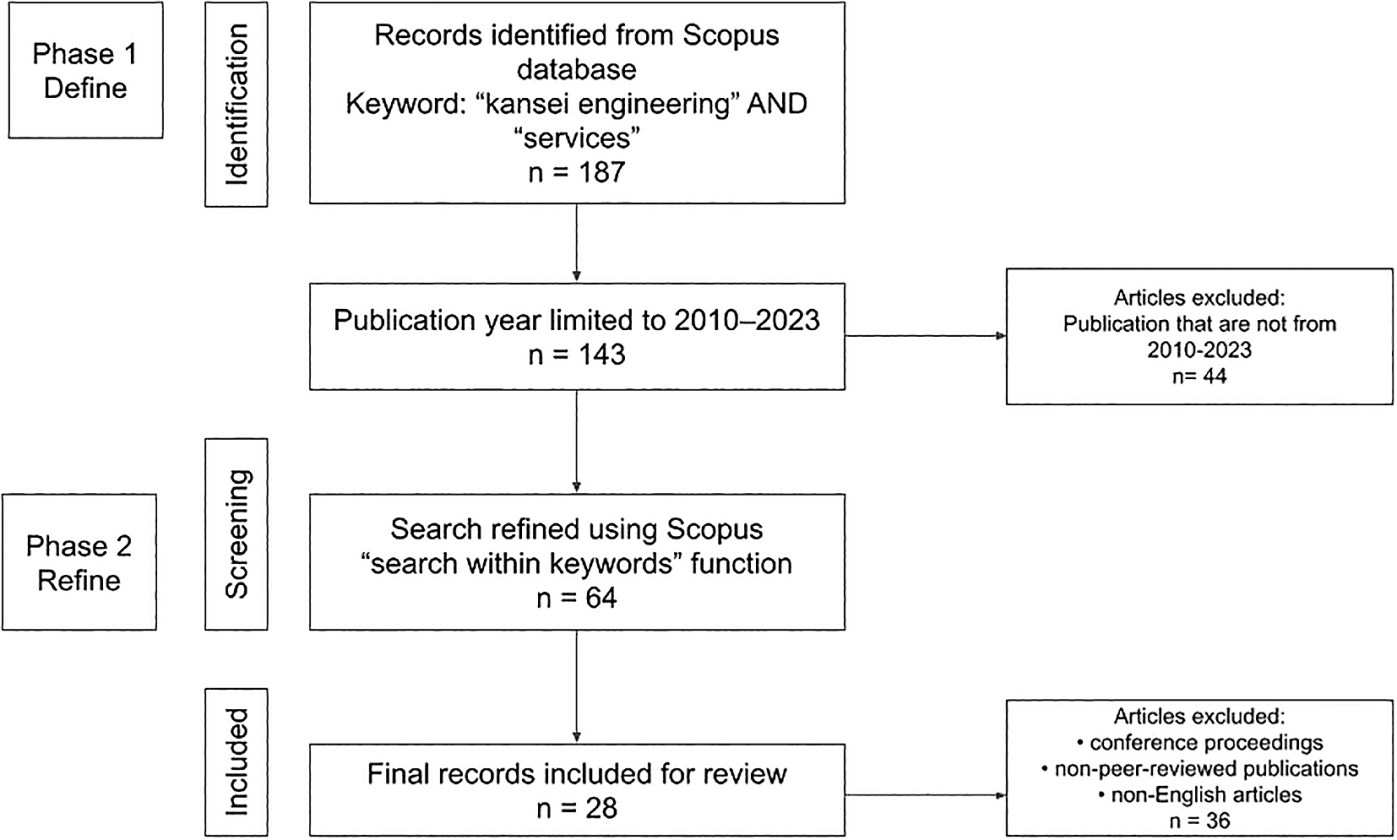
PRISMA-based flow diagram illustrating the two-phase Define–Refine literature review process for studies on Kansei Engineering (KE) applications in service contexts. The diagram shows the identification of records through the Scopus database (2010–2023), screening and refinement procedures, exclusion criteria, and the final inclusion of 28 peer-reviewed journal articles.

Methodological rigor was ensured through a two-phase “Define–Refine” filtering protocol, accompanied by an explicit consideration of potential biases such as single-database dependence, methodological heterogeneity, and the cultural concentration of studies in Asian contexts. The analytical approach integrated thematic synthesis, methodological clustering, cross-industry comparison, and concept mapping to develop consolidated theoretical insights. Phases of the review process are as follows: (1) Phase 1 (Define) and (2) Phase 2 (Refine) (see Figure 1). The review employed a structured search strategy using the Scopus database. The literature search was conducted using the Scopus database. Scopus was selected due to its extensive coverage of peer-reviewed journals across multiple disciplines, including engineering, design, and social sciences, making it particularly suitable for multidisciplinary topics such as KE in service contexts. As one of the largest curated abstract and citation databases, Scopus provides robust indexing, advanced search functionalities, and reliable citation tracking, which support transparency and reproducibility in literature reviews. The use of a single database is consistent with the semi-systematic review approach, which prioritizes conceptual breadth and interpretive synthesis over exhaustive retrieva. ^14^ However, it is acknowledged that limiting the search to Scopus may introduce database bias and potentially omit relevant studies indexed in other sources such as Web of Science or IEEE Xplore. Future research could incorporate multiple databases to enhance coverage and further validate the findings.

In Phase 1 (Define), an initial search was performed using the keywords “kansei engineering” AND “services”, yielding 187 records. The publication timeframe was then limited to 2010–2023, reducing the dataset to 143 records to reflect contemporary developments in KE research. The use of the term “services” in this initial phase was intended to capture the broad application domain of KE beyond product-oriented contexts, including areas such as service experience, customer interaction, and service systems.

In Phase 2 (Refine), the search was narrowed to improve topical specificity. A keyword-based filtering step was applied using Scopus’ “search within keywords” function, resulting in 64 records that more directly addressed service-related applications of KE. To ensure academic quality and consistency, inclusion criteria were applied to retain only peer-reviewed journal articles, book chapters, and review papers, while excluding conference proceedings, non-peer-reviewed publications, and non-English articles. This process resulted in a final dataset of 28 articles. Duplicate records were identified and removed manually through cross-checking of article titles, author names, publication years, and Digital Object Identifiers (DOIs), ensuring that all included studies were unique and eliminating potential redundancy in the dataset.

To ensure transparency and replicability in the search and selection process, the review procedure was documented using the PRISMA flow diagram. Although this study adopts a semi-systematic review approach, PRISMA guidelines were used as a reporting framework to clearly present the identification, screening, and inclusion stages of the literature selection process (see XI. Data Availability).

Data extraction was conducted systematically for each of the 28 selected studies to ensure consistency and transparency in the review process. A predefined extraction framework was developed prior to analysis, consisting of the following categories: (1) study reference, (2) research context or application domain, (3) methodological approach or models used (e.g., KE, Kano, SERVQUAL, QFD, machine learning), and (4) key findings related to KE in service settings. Each article was reviewed in full, and relevant information was recorded according to these categories and organised into a structured comparison table ( Table 1). This table enabled consistent cross-study comparison and supported the identification of recurring patterns across research contexts, analytical techniques, and service applications, which later informed the thematic clustering presented in the Results section.

**Table 1.** Summary of 28 selected studies on the application of Kansei Engineering (KE) in service and product–service contexts, outlining research settings, methodological approaches, and key findings related to emotional satisfaction, service quality, and design decision-making.

No	Reference	Context	Methods or models used and discussed	Main Findings and Theme
1	11	Luxury 4- & 5-star hotel services	General KE methodology, SERVQUAL, Kano model	This article discusses how the Kano model and KE can be used iteratively to improve service quality, focusing on how the performance level of service affects how the customers feel and experience. The Kano model categorizes service performance into three categories, i.e., must-be (M), one-dimensional (O), and attractive (A). The result of an empirical study of 100 respondents who stayed in premium 4- and 5-star hotels sheds light on which service items should be prioritized due to their strong impact on customer Kansei as a priority. Theme: KE for Service Quality Enhancement
2	14	Luxury hotel services	General KE methodology, Kano model, Markov chain	Customers today mostly prioritize fulfilling their emotional needs over functionality and usability, leading to the need for products and services to be more attractive and pleasing to customers' emotions. KE has been widely applied to explore customer emotions in design parameters, optimize properties not directly visible, and accommodate 21st-century trends, which are hedonism and more individualistic designs. This study focuses on service quality attributes as determinants of customer delight and loyalty, using Kano's model to show the relationship between service attribute performance and emotional response. The study surveyed luxury hotel services that focus on Singaporean and Indonesian tourists and found three key service attributes (i.e., visually clean outdoor environments, efficient employees, and consistent courteousness). The study offers several practical implications, including prioritizing efforts to improve service quality, providing guidelines for practitioners, and utilizing Markov chains to understand customer needs over time and prepare appropriate response strategies. Theme: KE for Service Quality Enhancement
3	15	Practical applications	General KE, service engineering, Bayesian network examination	KE and service engineering share similarities and differences. KE emphasizes individual value, using the covariance structure analytical method and ontology to construct a system structure. While Bayesian network examination is used for precise treatment, with practical applications provided. Theme: KE for Service Quality Enhancement
4	16	Online shopping services	General KE, International Affective Picture System (IAPS)	This study investigates the relationship between pupil size and user subjective opinion using International Affective Picture System (IAPS) images and product images. Participants have viewed scrambled and unscrambled types of images, and their affective response to the target image (Kansei) was recorded and analysed. The findings showed that IAPS images influenced different variations in pupil sizes, while product images showed larger variations. The results of the study support the claim that pupil size can be utilized to assess product images, potentially allowing online shopping service providers to measure customers' pupils' sizes to determine whether the products are liked or not. Theme: Behavioral and Psychophysiological KE
5	17	Home delivery service (HDS) industry	General KE	The home delivery service (HDS) industry has seen rapid growth due to increased internet and television shopping. To maintain a competitive edge, service providers must continuously improve and offer differentiation. Designers must capture customers' feelings to design new services that meet their expectations. KE, using Partial Least Square (PLS), can be used to quantify the relationship between customer emotions and HDS characteristics. This study provides an exemplification of applying KE to service design in service industries. Theme: KE for Service Quality Enhancement
6	12	International express services (IESs)	General KE	This paper analyses the relationship between service attributes and customer Kansei perceptions and usage intention in international express services (IESs) to gain a competitive advantage in the logistics market. Using KE methodology, the study identifies five important service attributes related to usage intention. The findings suggest that international express managers should prioritize service elements that elicit Kansei perceptions and lead to pre-purchase usage intention. The study also suggests incorporating missing Kansei perceptions from customers' post-purchase experiences into future design considerations. Theme: KE for Service Quality Enhancement
7	18	Hotel services	Path analysis	The study examines the mediating role of emotional and cognitive satisfaction in the relationship between quality of service and customer loyalty. This research involved 102 respondents from 24 hotels in Surabaya, Indonesia. Results indicated that overall customer satisfaction (known as cognition) and emotion (known as Kansei) partially mediated the relationship, with Kansei accounting for 24% (compared to 28% due to cognition) of the effect. The study's generalization is limited due to the small sample size. Theme: KE for Service Quality Enhancement
8	19	A medium-sized restaurant services	General KE, Kano model, Theory of Inventive Problem Solving (TRIZ)	This study proposes an integrative model of KE, the Kano model, and the Theory of Inventive Problem Solving (TRIZ) to capture customer emotions. The Kano model presents the relationship between service attribute performance and customer satisfaction, while TRIZ generates innovative designs for improvement. The study considers diverse cultural backgrounds to better understand the emotional needs of customers from different backgrounds. It utilizes an empirical study in a medium-sized restaurant to demonstrate the applicability of the integrated model. Theme: KE for Service Quality Enhancement
9	20	Cross-border logistics services (CBLS)	General KE, online content mining	This study highlights cross-border e-commerce that has increased the demand for cross-border logistics services (CBLS). To maintain a competitive value, providers must continually improve and differentiate their offerings. This study develops a customer-based loyalty system (CBLS) by applying the KE model to meet the emotional expectations of customers. To analyse customer feelings and service elements, this study utilizes the Partial Least Squares. Online content mining helps identify service elements and Kansei words. This study exemplifies the integration of KE and online content analysis in the service business sector. Theme: Data-Driven and Analytics-Based KE
10	21	Logistic services	General KE, quality function deployment (QFD)	With a range of 2004 to 2014, Indonesia's logistics sector experienced significant growth, necessitating a focus on overall customer satisfaction. While current studies primarily concentrate on service gaps, a deeper comprehension of customer affective needs (Kansei) is highly essential for gaining a competitive advantage. This study proposes a combined model of KE, Kano, and Quality Function Deployment (QFD) to generate innovative ideas for increasing customer emotional satisfaction and delight. To prove the applicability of the conceptual framework, this study conducted a case study in logistic services. Afterward, innovative strategies are proposed. Theme: KE for Service Quality Enhancement
11	22	Airline services	General KE, quality function deployment (QFD)	The aviation industry in Indonesia has been extensively expanded as a response to ASEAN Open Sky promoting liberalization. This study utilizes an integrated model of KE incorporating Quality Function Deployment (QFD) in identifying design service attributes that are influencing customer emotional satisfaction. It is expected to enhance the airline service quality by addressing attractive service attributes and Kansei words. Action plans were formulated, including airline alliances, brand identity, seat classes, modern information systems, and airline expert consultation. Theme: KE for Service Quality Enhancement
12	23	Door-to-door delivery (D2DD) services	General KE, data mining	An integrated model of KE incorporating data mining techniques, in this study, has been applied for door-to-door delivery (D2DD) service design and improvement. It collects and finalizes customers' Kansei words and identifies service properties. Afterwards, it quantifies the relationship between these properties, Kansei responses, and usage intention using a decision tree. The result highlights that a combination of key Kansei responses leads to positive customer usage intention, allowing providers to improve their service of excellence. Theme: Data-Driven and Analytics-Based KE
13	24	Product (a recliner)	General KE, text mining, self-organizing map (SOM)	This study aims to develop an affective variable extraction methodology for KE-based products. It extracted users' Kansei variables from online reviews and classified them using a self-organizing map (SOM). A product experiment on recliners was performed on Amazon.com . The results showed that comfort was the Kansei most frequently associated with the use of recliners. The study urges that text mining techniques and SOM can be used to analyse customers' Kansei effectively, and it will be enhancing understanding of their emotions regarding recliners. Theme: Data-Driven and Analytics-Based KE
14	25	Hotel services	General KE, text mining	This study has been conducted to formulate practical guidelines for hotel service development using an approach combining KE and text mining. The data of online customers’ reviews and feedback were utilized, as their opinions are deemed crucial for selecting particular hotels. This study uses text mining to extract Kansei words and hotel service characteristics from online content and generates relationships using link analysis. The findings provide a comprehensive understanding of customer feedback. Moreover, it provides strategies on how to increase customer satisfaction, differentiate products and services, and improve hotel performance. The study offers implications for both practice and theory. Theme: Data-Driven and Analytics-Based KE
15	26	Logistics service	General KE, logistics service quality	This study has been conducted to investigate the relationship between logistics service quality and customer satisfaction and loyalty among humanitarian logistics providers in Indonesia. It focuses on personnel, operations, and technological support. The findings support that service quality has a major impact on customer satisfaction. The study employs KE to gather customers’ emotional perceptions of the quality of relief logistics services, providing a unique perspective on the industry. Theme: KE for Service Quality Enhancement
16	27	Client feedback of product and service development	General KE, sentiment analysis	This study discusses how online reviews are critical for KE, that incorporates customer feedback into product and service improvement. KE is conducted in India and Pakistan using unstructured reviews. However, the language barrier prevents sentiment analysis. This study intends to conduct aspect-based sentiment analysis on these reviews and translate them into English. Common service features and attitudes were extracted, then clustered with unsupervised machine learning. Ridesharing businesses, as a result, will be continuously improved in response to customer expectations, thereby growing their business. Theme: Data-Driven and Analytics-Based KE
17	6	International airport lounge and lobby services	General KE, TRIZ (Theory of Inventive Problem Solving)	This study proposes a refined integrated method for measuring the impact of service performance equipped with Kano categories on emotional well-being (Kansei). Afterward, it comes up with new ideas for long-lasting service solutions using TRIZ. This study promotes KE by emphasizing the emotional satisfaction that customers experience because of their perception of the service. The study employs an empirical study of international airport lounge and lobby services, with a focus on Kansei's role in sustainable service development. The study recommends that service designers and managers promote attractive-based service features (which are related to the safety and security of the passengers) while keeping Kansei satisfaction in mind. Theme: KE for Service Quality Enhancement
18	28	Campus express delivery service	General KE, multinomial logistic regression, the Kano model, and Prospect Theory	This study proposes an uncertain KE methodology for customer behavioral-based service design. The delivery service attributes, emotional needs, and overall customer satisfaction were modelled to redesign services that meet customers' Kansei. This study utilizes logistic regression, the Kano model, and prospect theory to effectively model customer satisfaction functions. The methodology is successfully applied to a case study of a campus express delivery service in China, yielding consistent results and useful insights. Theme: Behavioral and Psychophysiological KE
19	29	Logistic services	General KE, SERVQUAL	The impact of logistics service quality on customer satisfaction and loyalty during the COVID-19 pandemic in the Indonesian context has been investigated. It has been found, in this study, that the prioritized effort will be on operational service, employee service, and technical service excellence. According to the study, staff and technical service quality have a significant impact on customer happiness, whereas customer trust has an important effect on customer loyalty. The use of Kansei brings along a unique viewpoint on customer service throughout the pandemic. Theme: KE for Service Quality Enhancement
20	30	Multi-application practice	CiteSpace’s visualisation analysis	KE has been used, in this study, to conduct research through Cite Space’s visualization techniques. There were 2830 Web of Science articles investigated, focusing on keywords such as knowledge sources, key contributions, interdisciplinary qualities, and major study topics. Research hotspots and cutting-edge technologies were identified, while also proposing upcoming trends, such as multidisciplinary collaboration, big data in the Internet era, and mathematical algorithm integration for multi-application practice. Theme: Data-Driven and Analytics-Based KE
21	31	Social and service robots	Systematic literature review	This study reviews the Kansei approach around creating social and service robots. It discusses the techniques, the concepts, the main types, and the objectives of eleven peer-reviewed publications. The study highlights that KE is an appropriate paradigm for robot design, allowing developers to comprehend design features for user acceptance. However, the application of KE approaches in robots remains underexplored, suggesting potential future paths and unanswered issues. Indeed, it is a big opportunity for deeper exploration of Kansei robot research. Theme: KE in Digital and Smart Service Systems
22	32	Service for colours of product	General KE, Search Neural Network, Convolutional Neural Network	This study has been done to highlight emotional-based design for product colour using KE methodology. It addresses a comprehensive relationship model between the colour of the product and the users’ emotional imagery through a search neural network and a convolutional neural network. When the system is applied to solve real-world design problems and issues, like designing a home service robot, it then provides relevant answers that satisfy the emotional image requirements for the customers or users. It is expected that the product colour emotional design theory will be applied widely. Theme: KE in Digital and Smart Service Systems
23	33	A soccer shoe design business model	General KE, Kawakida Jirou	The objective of this study is to create and demonstrate a recommendation system for Kansei soccer shoes that combines several technologies considering user’s Kansei (semantic needs), appearance design, and shoe-form categories. The system will analyse, evaluate, and categorize design features and attributes using KE incorporating factor analysis and 203 soccer shoe images. This recommendation system suggests soccer shoe samples that fit users, with an overall satisfaction rate of 87.08%. The findings highlight that developing a new business model by incorporating users’ Kansei requirements into shoe-form categories may potentially increase customer satisfaction and delight. Theme: KE in Digital and Smart Service Systems
24	34	The bionic design of a unmanned aerial vehicle (UAV) product	General KE, Biologically Inspired Design (BID)	It is to showcase KE to create a data-driven intelligent service model for biologically inspired design (BID), as highlighted in the study. It bridges the gap between customers and designers by wrapping up the perceptual characteristics of creatures, taken from product semantics. Based on user preferences, the model predicts biodata and executes a BID library. The study creates a computer-aided design service system for a bionic unmanned aerial vehicle (UAV) system, thereby enhancing design efficiency. This study solves the cognitive constraints and potential discrepancies between designers and users encourages the use of bioinspiration in product design, which can increase marketability. Theme: KE in Digital and Smart Service Systems
25	35	General logistics services in Indonesia	General KE, conjoint analysis	This study proposes an integrative model of KE and conjoint analysis to investigate customer preferences for logistics services in Indonesia. One hundred respondents from East Java, Indonesia, completed questionnaires, and thirty of them identified specific qualities. Key characteristics include delivery services, courier attitude, order information, product quality, and warehouse location. The most desirable attributes formulated are intact products and a courteous attitude among the staff. Theme: KE for Service Quality Enhancement
26	36	In-flight service of a Chinese airline	General KE, Kano, partial least squares algorithm, decision tree mining	This study proposes a combined framework of KE and the Kano model applied to service design that prioritizes customer-perceived preferences. The model utilizes the partial least squares method and decision tree mining to create a link between customer perception and service attributes, which influences customer emotional satisfaction. The study formalizes the intended proposed strategies for service design improvement and development. Theme: KE for Service Quality Enhancement
27	37	General product-service system (PSS)	Knowledge Graph (KG), Mass Personalization (MP)	This study used the product-service system incorporating the smart system (known as Smart PSS) configuration approach. To meet individualized and dynamic customer needs, it is then to obtain mass personalization (MP). The study utilizes a knowledge graph (KG) to achieve comprehensive system knowledge by combining field, design, and user data. This hybrid integrative approach addresses question answering with similarity calculation to provide relevant findings. The proposed configuration process will then be integrated into Smart PSS's reconfiguration lifecycle, which allows for dynamic adjustments. Theme: KE in Digital and Smart Service Systems
28	38	International airport services	General KE, robust design, SERVQUAL, Kano, TRIZ	KE is an important tool in service design, as it focuses on translating consumers' emotional demands into service qualities. However, its validity and robustness have been questioned due to the changing nature of passengers' requirements and service constraints. A more structured KE methodology, which includes Kansei text mining, is proposed for robust service design. The Taguchi method is used to refine the formulated strategy. Theme: Data-Driven and Analytics-Based KE

Although this study does not adopt a fully systematic review protocol, a basic quality appraisal was conducted to ensure the credibility of the included studies. The selected articles were evaluated based on three criteria: (1) methodological clarity, (2) analytical rigor, and (3) relevance to KE in service contexts. Studies demonstrating structured methodologies (e.g., statistical modelling, machine learning, or validated frameworks) were considered higher in rigor, while descriptive studies without validation were noted as lower in methodological strength.

## V. Results and discussion

Table 1 summarises the 28 selected studies on KE applications in services, including their research contexts, methodological approaches, and key findings.

Based on the synthesis of the 28 selected studies ( Table 1), a thematic analysis was conducted to identify recurring patterns in research objectives, methodological approaches, and application contexts of KE in service-related domains. We adopt a pattern-based clustering approach to reveal dominant directions and underlying structures within the literature.

The thematic clusters were derived through iterative comparison across studies, focusing on how KE is operationalised, integrated with other methods, and applied across different service and product–service contexts. This process resulted in four major thematic clusters: (1) KE for service quality enhancement (46%), (2) data-driven and analytics-based KE (25%), (3) KE in digital and smart service systems (18%), and (4) behavioral and psychophysiological KE (7%).

### A. KE for service quality enhancement

The largest proportion of the reviewed studies positions KE within the domain of service quality enhancement and prioritisation. In this cluster, KE is frequently integrated with established service quality and decision-support frameworks, such as SERVQUAL, the Kano model, Quality Function Deployment (QFD), and the Theory of Inventive Problem Solving (TRIZ). ^11, 22, 23, 37^

These integrations enable the systematic translation of customers’ emotional responses (Kansei) into structured service attributes and design parameters. Rather than treating emotions as abstract or subjective inputs, KE in this cluster functions as a bridging mechanism that connects affective perceptions with measurable service characteristics. ^18, 24^ This allows practitioners to identify which service attributes should be prioritised based on their impact on customer satisfaction, loyalty, and perceived quality. ^19, 30^

A consistent pattern across these studies is the emphasis on prioritisation under constraints, where KE is used to guide decision-making in environments with limited resources, such as logistics services, hospitality, and airline industries. However, many of these approaches tend to treat Kansei responses as relatively static, often relying on cross-sectional data without capturing the dynamic and evolving nature of customer emotions over time.

In addition, many studies extend this approach by linking Kansei responses to broader outcome variables, such as customer satisfaction, trust, loyalty, and behavioural intention. ^19, 27, 30^ This indicates a shift from descriptive emotional analysis toward more performance-oriented models, where emotional satisfaction is treated as a key mediator between service attributes and customer behaviour. As a result, KE becomes embedded within broader service evaluation frameworks, reinforcing its role as a decision-support tool rather than a purely exploratory method.

### B. Data-Driven and analytics-based KE

The second thematic cluster reflects a significant shift toward data-driven and analytics-based approaches in KE. In contrast to traditional survey-based methods, these studies employ advanced analytical techniques such as text mining, sentiment analysis, decision trees, Partial Least Squares (PLS), and big data analytics to extract and model customer emotional responses from large-scale datasets. ^21, 39, 28^

A defining characteristic of this cluster is the utilisation of user-generated data, particularly online reviews, as a primary source for capturing Kansei. Instead of relying solely on predefined semantic scales, these approaches analyse naturally occurring language data to identify emotional expressions and service attributes directly from customer experiences. ^25, 39^ This enables a more scalable and dynamic understanding of customer perception, especially in rapidly evolving service environments such as e-commerce, logistics, and digital platforms.

Furthermore, several studies extend this approach by integrating KE with machine learning and data mining techniques, such as self-organising maps (SOM), aspect-based sentiment analysis, and decision tree models, to uncover complex relationships between service attributes and emotional responses. ^25, 24, 28^ These methods allow for the identification of hidden patterns and clusters of affective experiences, supporting more nuanced segmentation of customer preferences and enabling data-driven service design decisions.

Another important development within this cluster is the incorporation of big data and online content analytics to enhance the robustness and validity of KE models. By analysing large volumes of real-time customer feedback, these studies aim to overcome limitations associated with small sample sizes and static survey instruments, providing more comprehensive insights into customer needs and expectations. ^21, 26^ This aligns with broader trends in design and service research, where data-driven approaches are increasingly used to support continuous improvement and adaptive service systems.

However, despite these advancements, several limitations remain. While data-driven KE approaches offer scalability and efficiency, they often rely on simplified representations of emotional responses, reducing complex affective experiences into categorical or polarity-based classifications. This may lead to a loss of contextual depth and overlook cultural, situational, and experiential nuances embedded in customer perceptions. Additionally, the reliance on digital trace data introduces challenges related to data quality, bias, and interpretability, particularly when dealing with multilingual or informal user-generated content. ^28^

### C. KE in Digital and smart service systems

The third thematic cluster highlights the application of KE within digital and smart service environments, reflecting the increasing convergence between affective design and intelligent systems. In this cluster, KE is not only used to analyse customer emotions but is increasingly embedded within digital platforms, automated systems, and intelligent service infrastructures to support adaptive and personalised service design. ^26, 38^

A key characteristic of this theme is the integration of KE with advanced computational technologies, including machine learning algorithms, neural networks, knowledge graphs, and recommendation systems. These approaches enable the development of intelligent systems that can dynamically interpret customer emotions and translate them into design solutions or service configurations in real time. ^33, 34^ For example, KE has been applied in AI-driven product and service design systems, where emotional preferences are linked to design parameters through deep learning models, allowing for automated generation of user-centred solutions. ^33^

In addition, several studies demonstrate the application of KE within smart product–service systems (Smart PSS), where both functional and affective requirements are considered simultaneously in system configuration and personalisation processes. ^38^ In such contexts, KE contributes to bridging the gap between user perception and system-level decision-making, enabling services to be continuously adjusted based on evolving user needs. Similarly, KE has been utilised in recommendation systems and digital platforms to enhance user experience by aligning service offerings with individual emotional preferences. ^34^

### D. Behavioral and Psychophysiological KE

The fourth thematic cluster focuses on behavioral and psychophysiological approaches to KE, representing a smaller yet conceptually significant direction within the literature. In contrast to the dominant reliance on self-reported data in KE studies, this cluster explores alternative methods for capturing emotional responses through objective measurements and behavioral modelling, aiming to reduce subjectivity and enhance the validity of Kansei evaluation. ^17, 29^

A key characteristic of this theme is the incorporation of physiological indicators, such as pupil size, as proxies for emotional arousal and affective response. These approaches attempt to capture unconscious or implicit emotional reactions that may not be fully expressed through questionnaires or interviews. ^17^ By measuring physiological responses during interaction with products or services, these studies provide an additional layer of insight into user experience, complementing traditional semantic and survey-based methods.

In parallel, several studies adopt behavioral modelling approaches to better understand the relationship between service attributes, emotional responses, and decision-making processes. For instance, probabilistic models, regression techniques, and frameworks incorporating behavioral theories (such as Prospect Theory) are used to address the uncertainty and nonlinearity inherent in customer emotions. ^29^ These approaches recognise that emotional responses are not always stable or rational, and attempt to model the variability, asymmetry, and bias present in human perception and evaluation.

This cluster therefore represents an effort to move beyond descriptive and static representations of Kansei toward a more dynamic and theoretically grounded understanding of affective experience. However, these approaches remain limited in scope and application. Many studies rely on controlled experimental settings with relatively small sample sizes, which may restrict their generalisability to real-world service environments. In addition, physiological measurements often require specialised equipment and controlled conditions, making them less practical for large-scale or industry-based applications. As a result, the integration of psychophysiological methods into mainstream KE practice remains at an early stage.

### E. Cross-Thematic insights and key limitations

**Figure 2.**
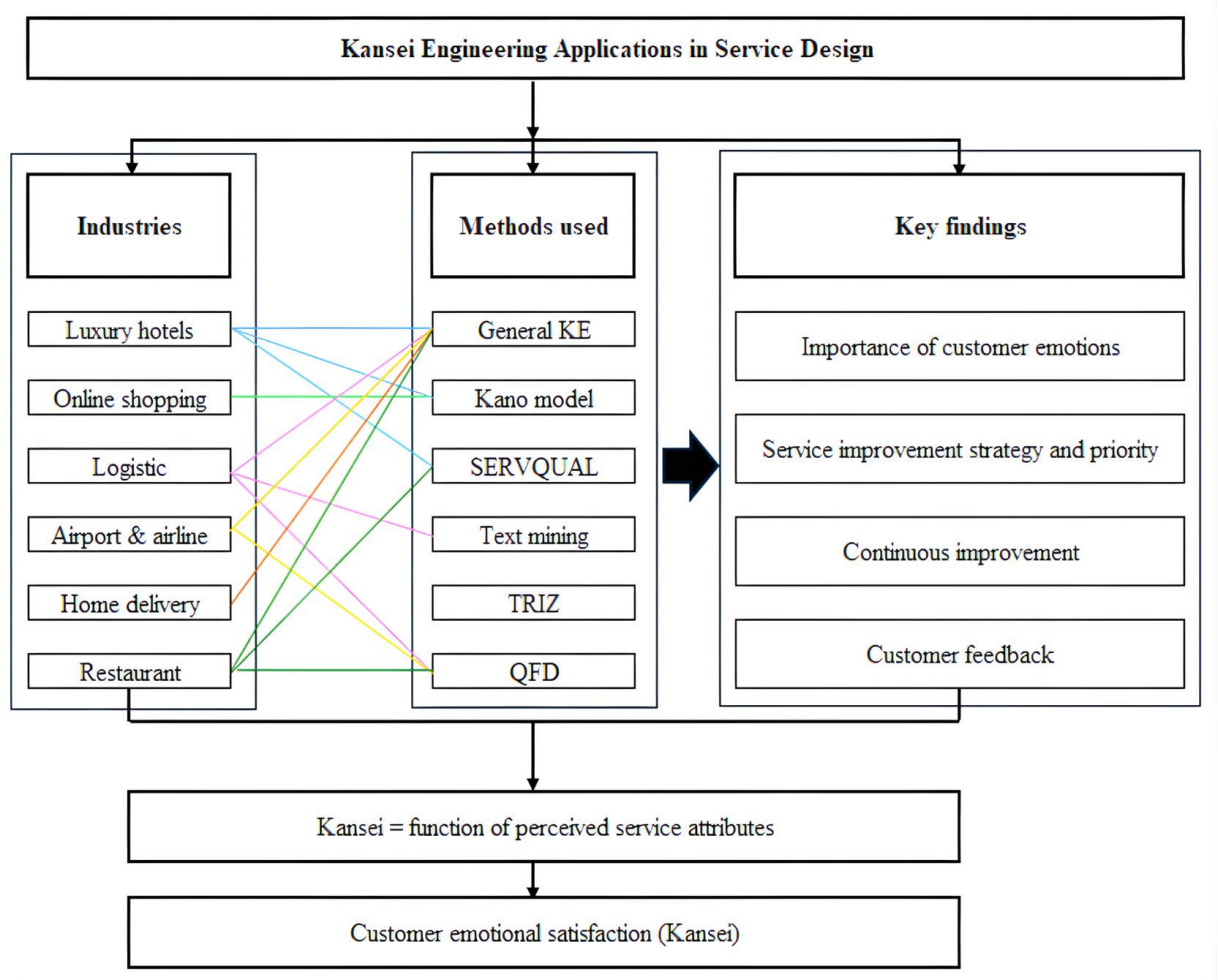
Framework of Kansei Engineering (KE) applications in service design. The framework illustrates the relationships between service industries, applied KE-related methods (General KE, Kano model, SERVQUAL, text mining, TRIZ, and QFD), and key findings. Colored connectors indicate the distribution of methodological applications across different service sectors. The framework highlights how Kansei Engineering functions as a mediator between perceived service attributes and customer emotional satisfaction (Kansei), informing service improvement strategies, prioritization, and continuous improvement.

One common finding among the studies is the use of KE to understand and measure emotional reactions and convert them into useful information for increasing customer happiness and loyalty. The main research domain in the literature review is KE, applied to service design for customer experience enhancement (see Figure 2). KE is often integrated with other methods and tools like the Kano Model, SERVQUAL, TRIZ, and data mining techniques to measure how customers feel about products and services in both numbers and descriptions. Inherently, this area of study is quite relevant in diverse industries, including logistics, hospitality, online services, retail, and healthcare. It is to emphasize how KE can adjust services to meet customer Kansei. In addition, this domain increasingly incorporates technologies such as sentiment analysis, text mining, and big data. For sure, it would be used to analyze user-generated content and gain real-time insights. This technological integration emphasizes an evolving trend within the KE domain toward more data-driven and automated approaches in digging for customer feedback and sentiment.

In terms of methodological divergence and reliability, a clear methodological gap emerges across the literature. At the higher end, research employing approaches such as PLS-SEM, logistic regression, and machine learning demonstrates stronger analytical rigor and validation. Mid-level studies often utilise integrated frameworks (e.g., KE–Kano–TRIZ), offering structured but partially subjective analysis. In contrast, descriptive KE studies frequently lack validation, triangulation, and cross-checking, limiting their reliability and comparability. This heterogeneity complicates the consolidation of findings and highlights the need for more consistent methodological standards in KE research.

Inherently, there are two major groups of reviewed publications of KE above, as follows. The first group is about KE application in the service industries. KE has been extensively applied in service industries, including logistics, hospitality, and retail, emphasizing its relevance in capturing customer experiences that are related to the fulfilment of their affective needs. The second one is the use of advanced analytical techniques and methods. Several research employ advanced data analysis methods and approaches such as partial least squares (PLS), text mining, and decision tree mining to analyze large datasets, especially from user-generated content like online reviews.

In terms of cross-industry insights, cross-industry evidence shows that KE delivers varying levels of effectiveness depending on sectoral characteristics. In hospitality and retail/e-commerce, KE performs strongly, revealing emotional touchpoints and capturing nuanced customer sentiments, though findings often depend heavily on Asian cultural contexts or online review data. Logistics and aviation display moderate to high effectiveness, as KE enhances perceptions of efficiency, trust, and service blueprinting, but these applications tend to underemphasize hedonic factors or lack longitudinal validation. Digital and robotic service contexts exhibit promising potential, yet empirical studies remain sparse. Recent healthcare research also highlights KE’s capacity to strengthen patient empathy and comfort, although the field was largely underexplored before 2023.

KE's research above differs in three key aspects. Based on diverse contexts and industries, some studies focus on traditional service industries (such as hotels, logistics, and higher education); others apply KE to more specific or emerging contexts, such as airport lounges, social robots, and in-flight services. This potential dynamic shows how flexible KE is. In addition, the KE research highlights that the findings might not always be applied to various industries directly. Some adjustments are needed. Theoretically, researchers who investigated KE have drawn on a variety of statistical techniques, including but not limited to knowledge graph techniques, sentiment analysis, structural equation modelling, fuzzy logic modelling, neural networks, and Bayesian network models. The technique selection may be influenced by the context of the study as well as the characteristics of the service settings under investigation. These methodological variations demonstrate how KE has progressively evolved, changed, and been modified over time to address various research issues and real-world applications. Regional and cultural focus factors have already been taken into consideration in service design by researchers studying logistics in East Asia or hospitality in Indonesia. These regional differences imply that cultural contexts and backgrounds may influence how people feel about certain aspects of services.

In terms of KE conceptual framework development, the proposed conceptual framework operates through a three-layer logic supported by a dynamic feedback loop. The input layer combines customers’ emotional needs expressed through Kansei words with contextual service attributes. These inputs flow into the processing layer, where insights are generated through traditional KE modeling, AI-enhanced KE techniques, or hybrid KE–quality approaches. The resulting outputs form the outcome layer, encompassing emotional satisfaction, behavioral intentions, loyalty, and differentiated service innovations. A feedback loop, powered by real-time analytics such as text mining, IoT data, and AI-based sensing, continuously updates the system to refine emotional alignment and enhance service performance over time.

There are potential future research directions for KE in service design. KE has promising potential to evolve alongside today’s prospective trends and technologies. New technologies like blockchain, artificial intelligence (AI), and the Internet of Things (IoT) might be considered for KE future research. It would create systems that are more reliable, responsive, adaptable, and transparent, including sustainable environments and autonomous service robots. Ways to measure sustainability in KE, making sure that service design is in line with eco-friendly practices, focusing on people, and cost-effectiveness will be highly promoted. Applying KE in more personalized services, utilizing AI-driven insights and big data to adjust services to individual Kansei in real time, can be regarded as an urgent call. Some human-based care industries, such as retail, healthcare, and hospitality, may gain advantages from such insights. Given the global services context, future research could investigate how KE frameworks will be applied to considering different cultural contexts. These results would bring benefits in the development of cross-culturally applicable universal KE guidance. Automated KE analysis systems that continuously process and interpret vast amounts of online reviews as user-generated content increases would be potentially offered to industries. Through this strategy, industries and practices could dynamically stay abreast of changing customer preferences, especially their Kansei. Future KE research with the long-term orientation, considering changes in customer loyalty and brand perception, could offer clues about how Kansei could be translated into sustained business success over time.

KE will face greater challenges in dynamic, data-driven, and culturally diverse contexts, contributing to the development of emotionally satisfying and sustainable services. One of the prominent future KE explorations is about healthcare services [39]. It is especially important to engage digital platforms in healthcare services. This category includes telemedicine platforms, mental health apps, wearable health monitors, and patient-centric online portals. Schütte et al. [10] have addressed similar topics. Their study addresses a comprehensive understanding of Kansei, a methodology using KE for healthcare services. Past relevant literature and industrial experience have been reviewed, providing the breadth of Kansei and its applications. The paper also includes a thematic mapping of the state-of-the-art and outlook, derived from interviews with 35 distinguished researchers. We find Kansei unique in its consideration of emotion in product design. The context of increasing information technology, digitalization, and possible integration with modern technologies like virtual reality (VR), artificial intelligence (AI), blockchains, and big data analytics has been discussed.

Nowadays, understanding and fulfilling the Kansei of patients, including the caregivers, is increasingly critical as hospital and healthcare services have become more patient-centered and more humanized. KE can contribute to the design of digital healthcare experiences that are not only functional but also emotionally appealing, making healthcare interactions more humane and impressive, especially in virtual settings where the lack of in-person interaction can feel impersonal. A comprehensive framework for designing digital health experiences, emphasizing the importance of addressing both functional and emotional aspects to enhance patient engagement, has been investigated [40]. Patients utilizing digital healthcare services often experience a wide range of emotions, including anxiety, trust, and hope. It leads to user adoption and continuation of use [41]. It is highly important how human-centered design principles are applied in e-mental health interventions, highlighting the role of empathy and user involvement in creating emotionally supportive digital health tools [42]. Here, KE can play its role to capture and translate patients’ Kansei into design parameters. Then, it leads to enhanced aspects like user interface comfort, reassuring communication, and intuitive interaction, which can importantly improve patient satisfaction and adherence to health interventions.

Wearable equipment, innovations in AI, and data-driven health platforms have been rapidly expanded in the digital healthcare sector. KE may play an important part in humanizing these technologies. Through IoT-based equipment such as wearable technology devices and real-time data collection, gathering prompt feedback on users’ Kansei can be distinctive. Practically, KE can utilize this data to continuously adapt services in real-time, creating a responsive healthcare environment adapted to individual emotional needs and preferences. The COVID-19 pandemic, for instance, has accelerated the adoption of digital healthcare, making telehealth a permanent fixture in healthcare delivery. Since telemedicine is becoming mainstream; as a matter of fact, KE can help address the unique Kansei associated with remote care, including creating feelings of trust and connection in a virtual setting.

The critical need to address patients’ Kansei, coupled with the growing potential for real-time data integration, makes digital hospital and healthcare service a promising field for KE studies. This context offers opportunities to strengthen KE methodologies by integrating the technological capabilities of modern healthcare platforms with empathy-driven cues. Considering Schutte et al.’s work [10], such advancements are particularly valuable in healthcare services, where patient comfort and Kansei are essential dimensions of service quality. For example, KE equipped with VR and AI could enable medical professionals to create more personalized and comforting environments for digital health services, such as telemedicine and virtual consultation platforms. These technologies can further maximize the Kansei experience during remote medical interactions by dynamically adapting to patients’ physiological and psychological data in real time.

In addition, the utilization of sentiment analysis and text mining on patient feedback, such as online reviews in customer-focused KE applications and healthcare services, can continuously refine digital platforms to address the patients’ emotional needs. More patient-centered, potentially leading to better health outcomes by encouraging positive impressions of treatment modes, will be a focus of such an approach in digital healthcare services. Future KE studies could promote VR and AI to monitor real-time users’ Kansei in digital healthcare, enabling proactive adjustments that instantly satisfy their emotional needs. In the digital age, this kind of application leads to more responsive and personalized service designs, indicating that KE has significant implications on shaping caring, high-quality digital healthcare services.

In terms of methodological divergence and reliability, a clear methodological gap emerges across the literature. At the high end, studies employing PLS-SEM, logistic regression, and machine-learning–based KE demonstrate strong analytical rigor and robust validation. Mid-level rigor appears in integrated KE–Kano–TRIZ frameworks, which offer structured analysis but rely on more qualitative or hybrid judgment. At the low end, descriptive KE studies often lack validation, triangulation, or cross-checking, limiting their reliability. This methodological heterogeneity weakens overall consistency and complicates meaningful comparison of findings across different industries and research contexts.

Inherently, there are two major groups of reviewed publications of KE above, as follows. The first group is about KE application in the service industries. KE has been extensively applied in service industries, including logistics, hospitality, and retail, emphasizing its relevance in capturing customer experiences that are related to the fulfilment of their affective needs. The second one is the use of advanced analytical techniques and methods. Several research employ advanced data analysis methods and approaches such as partial least squares (PLS), text mining, and decision tree mining to analyze large datasets, especially from user-generated content like online reviews.

In terms of cross-industry insights, cross-industry evidence shows that KE delivers varying levels of effectiveness depending on sectoral characteristics. In hospitality and retail/e-commerce, KE performs strongly, revealing emotional touchpoints and capturing nuanced customer sentiments, though findings often depend heavily on Asian cultural contexts or online review data. Logistics and aviation display moderate to high effectiveness, as KE enhances perceptions of efficiency, trust, and service blueprinting, but these applications tend to underemphasize hedonic factors or lack longitudinal validation. Digital and robotic service contexts exhibit promising potential, yet empirical studies remain sparse. Recent healthcare research also highlights KE’s capacity to strengthen patient empathy and comfort, although the field was largely underexplored before 2023.

KE's research above differs in three key aspects. Based on diverse contexts and industries, some studies focus on traditional service industries (such as hotels, logistics, and higher education); others apply KE to more specific or emerging contexts, such as airport lounges, social robots, and in-flight services. This potential dynamic shows how flexible KE is. In addition, the KE research highlights that the findings might not always be applied to various industries directly. Some adjustments are needed. Theoretically, researchers who investigated KE have drawn on a variety of statistical techniques, including but not limited to knowledge graph techniques, sentiment analysis, structural equation modelling, fuzzy logic modelling, neural networks, and Bayesian network models. The technique selection may be influenced by the context of the study as well as the characteristics of the service settings under investigation. These methodological variations demonstrate how KE has progressively evolved, changed, and been modified over time to address various research issues and real-world applications. Regional and cultural focus factors have already been taken into consideration in service design by researchers studying logistics in East Asia or hospitality in Indonesia. These regional differences imply that cultural contexts and backgrounds may influence how people feel about certain aspects of services.

In terms of KE conceptual framework development, the proposed conceptual framework operates through a three-layer logic supported by a dynamic feedback loop. The input layer combines customers’ emotional needs expressed through Kansei words with contextual service attributes. These inputs flow into the processing layer, where insights are generated through traditional KE modeling, AI-enhanced KE techniques, or hybrid KE–quality approaches. The resulting outputs form the outcome layer, encompassing emotional satisfaction, behavioral intentions, loyalty, and differentiated service innovations. A feedback loop, powered by real-time analytics such as text mining, IoT data, and AI-based sensing, continuously updates the system to refine emotional alignment and enhance service performance over time.

There are potential future research directions for KE in service design. KE has promising potential to evolve alongside today’s prospective trends and technologies. New technologies like blockchain, artificial intelligence (AI), and the Internet of Things (IoT) might be considered for KE future research. It would create systems that are more reliable, responsive, adaptable, and transparent, including sustainable environments and autonomous service robots. Ways to measure sustainability in KE, making sure that service design is in line with eco-friendly practices, focusing on people, and cost-effectiveness will be highly promoted. Applying KE in more personalized services, utilizing AI-driven insights and big data to adjust services to individual Kansei in real time, can be regarded as an urgent call. Some human-based care industries, such as retail, healthcare, and hospitality, may gain advantages from such insights. Given the global services context, future research could investigate how KE frameworks will be applied to considering different cultural contexts. These results would bring benefits in the development of cross-culturally applicable universal KE guidance. Automated KE analysis systems that continuously process and interpret vast amounts of online reviews as user-generated content increases would be potentially offered to industries. Through this strategy, industries and practices could dynamically stay abreast of changing customer preferences, especially their Kansei. Future KE research with the long-term orientation, considering changes in customer loyalty and brand perception, could offer clues about how Kansei could be translated into sustained business success over time.

KE will face greater challenges in dynamic, data-driven, and culturally diverse contexts, contributing to the development of emotionally satisfying and sustainable services. One of the prominent future KE explorations is about healthcare services. ^39^ It is especially important to engage digital platforms in healthcare services. This category includes telemedicine platforms, mental health apps, wearable health monitors, and patient-centric online portals. Schütte et al. ^10^ have addressed similar topics. Their study addresses a comprehensive understanding of Kansei, a methodology using KE for healthcare services. Past relevant literature and industrial experience have been reviewed, providing the breadth of Kansei and its applications. The paper also includes a thematic mapping of the state-of-the-art and outlook, derived from interviews with 35 distinguished researchers. We find Kansei unique in its consideration of emotion in product design. The context of increasing information technology, digitalization, and possible integration with modern technologies like virtual reality (VR), artificial intelligence (AI), blockchains, and big data analytics has been discussed.

Nowadays, understanding and fulfilling the Kansei of patients, including the caregivers, is increasingly critical as hospital and healthcare services have become more patient-centered and more humanized. KE can contribute to the design of digital healthcare experiences that are not only functional but also emotionally appealing, making healthcare interactions more humane and impressive, especially in virtual settings where the lack of in-person interaction can feel impersonal. A comprehensive framework for designing digital health experiences, emphasizing the importance of addressing both functional and emotional aspects to enhance patient engagement, has been investigated. ^40^ Patients utilizing digital healthcare services often experience a wide range of emotions, including anxiety, trust, and hope. It leads to user adoption and continuation of use. ^41^ It is highly important how human-centered design principles are applied in e-mental health interventions, highlighting the role of empathy and user involvement in creating emotionally supportive digital health tools. ^42^ Here, KE can play its role to capture and translate patients’ Kansei into design parameters. Then, it leads to enhanced aspects like user interface comfort, reassuring communication, and intuitive interaction, which can importantly improve patient satisfaction and adherence to health interventions.

Wearable equipment, innovations in AI, and data-driven health platforms have been rapidly expanded in the digital healthcare sector. KE may play an important part in humanizing these technologies. Through IoT-based equipment such as wearable technology devices and real-time data collection, gathering prompt feedback on users’ Kansei can be distinctive. Practically, KE can utilize this data to continuously adapt services in real-time, creating a responsive healthcare environment adapted to individual emotional needs and preferences. The COVID-19 pandemic, for instance, has accelerated the adoption of digital healthcare, making telehealth a permanent fixture in healthcare delivery. Since telemedicine is becoming mainstream; as a matter of fact, KE can help address the unique Kansei associated with remote care, including creating feelings of trust and connection in a virtual setting.

The critical need to address patients’ Kansei, coupled with the growing potential for real-time data integration, makes digital hospital and healthcare service a promising field for KE studies. This context offers opportunities to strengthen KE methodologies by integrating the technological capabilities of modern healthcare platforms with empathy-driven cues. Considering Schutte et al.’s work, ^10^ such advancements are particularly valuable in healthcare services, where patient comfort and Kansei are essential dimensions of service quality. For example, KE equipped with VR and AI could enable medical professionals to create more personalized and comforting environments for digital health services, such as telemedicine and virtual consultation platforms. These technologies can further maximize the Kansei experience during remote medical interactions by dynamically adapting to patients’ physiological and psychological data in real time.

In addition, the utilization of sentiment analysis and text mining on patient feedback, such as online reviews in customer-focused KE applications and healthcare services, can continuously refine digital platforms to address the patients’ emotional needs. More patient-centered, potentially leading to better health outcomes by encouraging positive impressions of treatment modes, will be a focus of such an approach in digital healthcare services. Future KE studies could promote VR and AI to monitor real-time users’ Kansei in digital healthcare, enabling proactive adjustments that instantly satisfy their emotional needs. In the digital age, this kind of application leads to more responsive and personalized service designs, indicating that KE has significant implications on shaping caring, high-quality digital healthcare services.

## VI. Limitations of the study

This review is subject to several limitations. First, reliance on a single database may have excluded relevant KE studies indexed in sources such as WoS or IEEE Xplore. The review focuses on a decade period of publications that are missing previous foundational studies on KE. Second, the strong cultural concentration of research, primarily from East and Southeast Asia, may introduce interpretive bias in Kansei constructs. Third, the heterogeneity of methodological approaches limits the feasibility of conducting meta-analyses. Fourth, the lack of empirical triangulation across studies constrains the validation of theoretical claims. Finally, temporal shifts in post-pandemic customer behavior are not fully reflected in pre-2020 KE studies, reducing their current applicability. Additionally, the study does not consider the longitudinal impact of KE implementations on service quality, making it difficult to assess their sustainability over time.

## VII. Conclusion and future research direction

A semi-systematic review of the development and application of KE in the service sector over the past decade (2010–2023) has been conducted through this study. This review concludes that KE in services has advanced from a descriptive emotional design tool to a data-enabled approach that strengthens service quality, customer experience, and digital transformation, positioning KE as a bridge to next-generation service innovation.

In the future study of service innovation, KE could potentially gain benefits. First, the integration of KE with intelligent technologies can be explored, such as AI and the Internet of Things (IoT), to enable real-time Kansei detection and adaptive service user interfaces. Second, as sustainability arises, KE can be integrated for the creation of services that are both environmentally responsible and emotionally engaging. Third, KE is crucial for mental health service quality improvement and digital healthcare, particularly as the request for more empathetic, trusted, and more human-centered virtual care rises. Fourth, by developing cross-cultural frameworks that differentiate universal from culture-specific emotional vocabularies and conducting longitudinal studies to capture evolving Kansei–loyalty relationships and post-pandemic emotional shifts.

## Data Availability

The PRISMA 2020 checklist and flow diagram supporting the reporting of this semi-systematic review are publicly available in the Figshare repository: https://doi.org/10.6084/m9.figshare.30999085 ^44^ The dataset is licensed under the CC BY 4.0 Universal license. This study is a semi-systematic literature review and does not generate primary quantitative or qualitative datasets. All data supporting the findings of this study are derived from previously published articles, which are fully cited in the Reference list. No new numerical datasets, raw values, participant-level data, or variables were generated or analysed in this review beyond the synthesis and interpretation of published literature.
