# Supplementary material for: Kansei Engineering in the Evolving Service Sector: A Decade of Insights
Source: F1000Res. 2026 Jun 3;15:301. Originally published 2026 Feb 20. [Version 2] doi: 10.12688/f1000research.174681.2 (PMC13324357; doi:10.12688/f1000research.174681.2)
Supplement: Peer review history

# Reviewer report: Reviewer response for version 2

**DOI:** 10.5256/f1000research.202143.r492240

**Published:** 2026-07-01

**Authors:** Shigekazu Ishihara

**Affiliations:** 1 Hiroshima International University, Hiroshima, Japan

The reviewer has recognized the authors' effort in applying Kansei Engineering for various service sectors. They published many applications and original papers on those topics. This paper is a review of research applying KE to the service sector, including the authors' and other researchers' work.

 The review is well structured according to the PRISMA guidelines. Reviewing other researchers' work is fair and well consolidated.

 The reviewer noted that the statistical analysis is insufficient. Those studies are published mainly in the disciplines of Management. Unlike in Medical Science research, there is a lack of computing and notes on Effect Size. Thus, meta-analytic methods are difficult to apply in this review. Despite this difficulty, the reviewer hopes for some quantitative analysis of the effect of applying KE. (This is not the condition of acceptance)

Are the rationale for, and objectives of, the Systematic Review clearly stated?

Yes

Is the statistical analysis and its interpretation appropriate?

Partly

If this is a Living Systematic Review, is the ‘living’ method appropriate and is the search schedule clearly defined and justified? (‘Living Systematic Review’ or a variation of this term should be included in the title.)

Yes

Are sufficient details of the methods and analysis provided to allow replication by others?

Yes

Are the conclusions drawn adequately supported by the results presented in the review?

Yes

Reviewer Expertise:

Kansei Engineering, Ergonomics, Gerontechnology, Applied Psychology.

I confirm that I have read this submission and believe that I have an appropriate level of expertise to confirm that it is of an acceptable scientific standard.

# Reviewer report: Reviewer response for version 2

**DOI:** 10.5256/f1000research.202143.r490735

**Published:** 2026-06-09

**Authors:** Priyakrushna Mohanty

**Affiliations:** 1 Christ University, Bengaluru, India

I feel the manuscript has improved substantially. However, I have a fair degree of apprehension regarding the study selection part. But, yes (may be) the use of semi systematic literature review permits the use of scholars' interpretation. Thereby, I recommend indexing.

Are the rationale for, and objectives of, the Systematic Review clearly stated?

Partly

Is the statistical analysis and its interpretation appropriate?

Not applicable

If this is a Living Systematic Review, is the ‘living’ method appropriate and is the search schedule clearly defined and justified? (‘Living Systematic Review’ or a variation of this term should be included in the title.)

Not applicable

Are sufficient details of the methods and analysis provided to allow replication by others?

No

Are the conclusions drawn adequately supported by the results presented in the review?

Partly

Reviewer Expertise:

Tourism, Aviation, SLR, Events Tourism, PLS-SEM

I confirm that I have read this submission and believe that I have an appropriate level of expertise to confirm that it is of an acceptable scientific standard.

# Response

**Published:** 2026-06-09

**Authors:** Markus Hartono

Dear Reviewer,

 Thank you very much for your positive review and recommendation. We appreciate your concern regarding the study selection process and have updated the paper to describe more clearly the search strategy, selection criteria and screening procedure. In addition, we have clarified the role of scholarly interpretation embedded in the semi-systematic review approach to enhance transparency and methodological rigor. Once again, we would like to thank you a lot on behalf of the authors.

# Reviewer report: Reviewer response for version 1

**DOI:** 10.5256/f1000research.192604.r472927

**Published:** 2026-04-21

**Authors:** Uma Pandey

**Affiliations:** 1 Jagran Lakecity University, Bhopal, Madhya Pradesh, India

1. The manuscript alternates between “Kansei Engineering (KE) ” and “KE” inconsistently. Define once and use consistently thereafter.

 2. The literature review about Kansei engineering includes relatively few citations, limiting its depth and coverage. Expanding the range of recent and relevant sources would strengthen the study’s credibility and comprehensiveness.

 3. The description of the screening stage requires clarification and improved precision. While the manuscript states that 63 records proceeded to screening and 35 were subsequently excluded, it does not clearly explain how this stage differs from the earlier refinement phase, creating ambiguity in the selection process. The inclusion and exclusion criteria are also limited, focusing primarily on publication type and language, without sufficient detail on how relevance to the study topic was assessed. Additionally, the statement “We completed 28 unique articles” is unclear and redundant, as it repeats information already provided. Overall, this section would benefit from clearer wording, removal of repetition, and a more detailed explanation of the screening procedures to enhance transparency and rigor.

 4. The authors should ensure that conclusions are more closely aligned with the presented results by avoiding broad generalizations and clearly linking claims to specific evidence from the reviewed studies. It is recommended to adopt more cautious language and consider variations in study quality and context when interpreting the findings. For example, instead of stating *“Kansei Engineering improves customer satisfaction and loyalty,”* the authors could revise this to *“The reviewed studies suggest that Kansei Engineering may contribute to improved customer satisfaction and loyalty, although the strength of this relationship varies across contexts and methodologies.”*

*5. *The authors should provide more detailed methodological information to enhance replicability, including the full search strategy (e.g., exact search strings and filters), a clearer description of the screening and selection process, and explicit details on data extraction and analysis procedures. Including these elements would improve transparency and allow other researchers to reproduce the study more reliably.

 6. The rationale and objectives are generally stated; however, they would benefit from greater clarity and specificity. The authors are encouraged to articulate more focused research questions and strengthen the rationale by clearly highlighting the key gaps in the literature that the review aims to address.

 For example, instead of broadly stating that *“Kansei Engineering has been applied in services,”* the authors could strengthen the rationale by writing: *“Despite the growing application of Kansei Engineering in service contexts, existing studies remain fragmented, with limited integration across theoretical frameworks and inconsistent methodological approaches, necessitating a comprehensive review.”*

Are the rationale for, and objectives of, the Systematic Review clearly stated?

Partly

Is the statistical analysis and its interpretation appropriate?

Not applicable

If this is a Living Systematic Review, is the ‘living’ method appropriate and is the search schedule clearly defined and justified? (‘Living Systematic Review’ or a variation of this term should be included in the title.)

Not applicable

Are sufficient details of the methods and analysis provided to allow replication by others?

Partly

Are the conclusions drawn adequately supported by the results presented in the review?

Partly

Reviewer Expertise:

Tourism, hospitality, marketing, gender studies, heritage

I confirm that I have read this submission and believe that I have an appropriate level of expertise to confirm that it is of an acceptable scientific standard, however I have significant reservations, as outlined above.

# Response

**Published:** 2026-05-19

**Authors:** Markus Hartono

Dear Dr. Uma Pandey,

 Thank you very much for your valuable feedback and constructive reviews. We sincerely appreciate the time and effort dedicated by the reviewers and the editorial team in evaluating our work.

 In response to the comments and suggestions provided, we have carefully revised the manuscript and addressed all reviewer feedback to the best of our ability. The revised manuscript, along with a detailed point-by-point response to the reviewers, has been uploaded via the following Google Drive link:

 https://drive.google.com/drive/folders/1FU1sDH5dumYr7yl50Z7UYraeOFdhmc8g

 We hope that the revisions have improved the clarity and overall quality of the manuscript. Thank you once again for your insightful comments and guidance throughout the review process.

 Sincerely,

 Markus Hartono

 On behalf of all authors

# Response

**Published:** 2026-05-19

**Authors:** Christabel Parung

**Affiliations:** Universitas Surabaya, Surabaya, East Java, Indonesia

Dear Dr. Uma Pandey,

 Thank you very much for your valuable feedback and constructive reviews. We sincerely appreciate the time and effort dedicated by the reviewers and the editorial team in evaluating our work.

 In response to the comments and suggestions provided, we have carefully revised the manuscript and addressed all reviewer feedback to the best of our ability. The revised manuscript, along with a detailed point-by-point response to the reviewers, has been uploaded via the following Google Drive link:

 https://drive.google.com/drive/folders/1FU1sDH5dumYr7yl50Z7UYraeOFdhmc8g

 We have already responded to these reviews in the system. However, the comments were not displayed. 

 We hope that the revisions have improved the clarity and overall quality of the manuscript. Thank you once again for your insightful comments and guidance throughout the review process.

 Sincerely,

 Christabel Parung 

 On behalf of all authors

# Reviewer report: Reviewer response for version 1

**DOI:** 10.5256/f1000research.192604.r472923

**Published:** 2026-04-09

**Authors:** Priyakrushna Mohanty

**Affiliations:** 1 Christ University, Bengaluru, India

**The review is compiled in three segments i.e. Section,** **Review Comments,** **Substantiation**

**Title**

 The title identifies the study as a Systematic Review, though the methodology section identifies it as a semi-systematic review. This poses theoretical confusion in terms of the type of review and rigor. The research does not provide a clear rationale behind the use of a semi-systematic approach and the need to still use PRISMA procedures.

 “A **semi-systematic literature review was conducted** using a two-phase Define-Refine protocol.”

**Abstract - Methods**

 There is limited transparency of the methods used in the abstract. It is not described in terms of essential SLR elements like search string, inclusion criteria, and synthesis method. It merely mentions database, timeframe and final sample size briefly.

 “A structured search was performed in the **Scopus database covering publications from 2010 to 2023**… resulting in the selection of **28 peer-reviewed journal articles**.”

**Introduction**

 The introduction is not clearly positioned to the existing review literature. It purports to have no systematic studies, but fails to show the gap in the review systematically using previous review studies or bibliometric data.

 “However, **there isn't a comprehensive study or paper that enumerates, organizes, and assesses all relevant work related to recommended KE frameworks**.”

**Research Gap Framing**

 The statements of research gaps seem description-based as opposed to analytical based on the previous literature. Systematic mapping of previous studies does not support them.

 “This review addresses three significant gaps in Knowledge Engagement (KE) literature within service contexts…”

**Review Type Justification**

 There is a poor rationale of applying a semi-systematic review. The claim that systematic reviews can overlook the pertinent studies is a speculation that has no methods references.

 “KE research spans engineering, design, psychology, and service science; therefore, **a rigid systematic protocol would exclude theoretically relevant studies**.”

**Search Strategy**

 The search strategy is very limited as it uses two keywords only. This can expose one to missing a significant chunk of the applicable literature in which KE can be talked about without the specific word service. There is no synonyms or Boolean expansion.

 “The initial search used the keywords **‘Kansei Engineering’ AND ‘services’**.”

**Database Selection**

 Only Scopus is used in the study, which reduces coverage and causes bias in databases. Within the engineering-related fields, major SLR guidelines suggest using multi-database searches (Scopus, WoS, IEEE, etc.).

 “A structured search was conducted in the **Scopus database**.”

**Search String Transparency**

 The entire search query, field limiters, and document filters are not mentioned in the paper. The study can not be replicated without this information.

 “The initial search used the keywords ‘Kansei Engineering’ AND ‘services’.”

**Search Refinement Process**

 Define-Refine protocol is largely eluded and seems arbitrary. The inclusion of the term service design at the refinement phase might be used inadvertently to omit any study that may be discussing KE in services but may not be using this term.

 “The keyword **‘service design’ was added to the initial search string**, resulting in a refined dataset of 63 records.”

**Screening Criteria**

 The inclusion and exclusion criteria are not complete. Some of the exclusion criteria are enumerated in the paper, but the inclusion criteria are not clearly defined.

 “Studies were excluded if they met any of the following criteria: (1) conference publications, (2) non-peer-reviewed journal articles, or (3) non-English publications.”

**Duplicate Screening**

 The manuscript says that it has no duplicates, but does not state how duplicates were identified and eliminated.

 “We completed **28 unique articles, with no duplicates**.”

**PRISMA Application**

 The authors state that they have adhered to PRISMA, but the key elements of the study, including quality assessment, bias assessment, and coding procedures of the study, are absent.

 “We used **PRISMA flow diagram to present the process** and the PRISMA 2020 checklist was also used.”

**Quality Assessment of Studies**

 The most significant methodological weakness is that quality appraisal of the included studies is not present. SLR standards usually have appraisal instruments (e.g., CASP, AMSTAR or home-made scoring schemes).

 No section describing quality assessment appears in the methodology.

**Data Extraction Protocol**

 The manner in which information was extracted in studies and multiple reviewers have not been described in the manuscript hence raising concerns of bias and reliability.

 The paper directly states: “Table 1 summarises the **28 selected studies**…” without describing extraction procedures.

**Synthesis Method**

 The synthesis method is vaguely outlined. Such words like thematic synthesis and methodological clustering are used without description of coding procedures and analytical frameworks.

 “The analytical approach integrated **thematic synthesis, methodological clustering, cross-industry comparison, and concept mapping**.”

**Thematic Analysis Validity**

 The methodology of obtaining the four thematic clusters is not clear. Coding rules, inter-coder reliability and clustering methodology are not described.

 “Analysis of 28 studies revealed **four thematic clusters** illustrating the diverse applications of KE.”

**Data Representation**

 Table 1 is not analytical but describes the situation. It abstracts the findings of studies without presenting systematic comparison measures of research design, sample size or theoretical background.

 “Table 1 summarises the **28 selected studies… including their research contexts, methodological approaches, and key findings**.”

**Conceptual Framework Development**

 The suggested framework is deductive and conceptual and not brought out clearly based on the review synthesis. The connection between themes extracted and framework components is not clearly shown.

 “The proposed conceptual framework operates through a **three-layer logic supported by a dynamic feedback loop**.”

**Results-Methodology Alignment**

 The results section is a combination of narrative discussion and interpretation, as opposed to structured synthesis (e.g., thematic matrices, frequency analysis, or bibliometric indicators).

 “Based on the summary of KE research… there are **differences, similarities, and potential areas for future research directions**.”

**Limitations Section**

 Although, the authors do point out a number of limitations, these limitations actually represent inherent flaws of the review methodology and not minor limitations.

 “First, **reliance on a single database** may have excluded relevant KE studies…”

**Reproducibility**

 The study cannot be replicated, which is a major requirement of systematic reviews, because of incomplete reporting of the search string, screening protocol, and coding procedure.

 The methodology only provides brief procedural statements without detailed protocols.

Are the rationale for, and objectives of, the Systematic Review clearly stated?

Partly

Is the statistical analysis and its interpretation appropriate?

Not applicable

If this is a Living Systematic Review, is the ‘living’ method appropriate and is the search schedule clearly defined and justified? (‘Living Systematic Review’ or a variation of this term should be included in the title.)

Not applicable

Are sufficient details of the methods and analysis provided to allow replication by others?

No

Are the conclusions drawn adequately supported by the results presented in the review?

Partly

Reviewer Expertise:

Tourism, Aviation, SLR, Events Tourism, PLS-SEM

I confirm that I have read this submission and believe that I have an appropriate level of expertise to state that I do not consider it to be of an acceptable scientific standard, for reasons outlined above.

# Response

**Published:** 2026-05-19

**Authors:** Christabel Parung

**Affiliations:** Universitas Surabaya, Surabaya, East Java, Indonesia

Dear Priyakrushna Mohanty, 

 Thank you very much for your valuable feedback and constructive reviews. We sincerely appreciate the time and effort dedicated by the reviewers and the editorial team in evaluating our work.

 In response to the comments and suggestions provided, we have carefully revised the manuscript and addressed all reviewer feedback to the best of our ability. The revised manuscript, along with a detailed point-by-point response to the reviewers, has been uploaded via the following Google Drive link:

 https://drive.google.com/drive/folders/1FU1sDH5dumYr7yl50Z7UYraeOFdhmc8g

 We hope that the revisions have improved the clarity and overall quality of the manuscript. Thank you once again for your insightful comments and guidance throughout the review process.

 Sincerely,

 Christabel Parung

 On behalf of all authors

# Response

**Published:** 2026-05-19

**Authors:** Markus Hartono

Dear Dr. Priyakrushna Mohanty,

 Thank you very much for your valuable feedback and constructive reviews. We sincerely appreciate the time and effort dedicated by the reviewers and the editorial team in evaluating our work.

 In response to the comments and suggestions provided, we have carefully revised the manuscript and addressed all reviewer feedback to the best of our ability. The revised manuscript, along with a detailed point-by-point response to the reviewers, has been uploaded via the following Google Drive link:

 https://drive.google.com/drive/folders/1FU1sDH5dumYr7yl50Z7UYraeOFdhmc8g

 We hope that the revisions have improved the clarity and overall quality of the manuscript. Thank you once again for your insightful comments and guidance throughout the review process.

 Sincerely,

 Markus Hartono

 On behalf of all authors
